# Improving Mental Health and Well-Being Through the Paradym App: Quantitative Study of Real-World Data

**DOI:** 10.2196/68031

**Published:** 2025-01-23

**Authors:** Athina Marina Metaxa, Shaun Liverpool, Mia Eisenstadt, John Pollard, Courtney Carlsson

**Affiliations:** 1 Nuffield Department of Primary Care Health Sciences University of Oxford Oxford United Kingdom; 2 Faculty of Health, Social Care and Medicine Edge Hill University Ormskirk United Kingdom; 3 Evidence Based Practice Unit Anna Freud National Centre for Children and Families University College London London United Kingdom; 4 Paradym London United Kingdom

**Keywords:** well-being, awareness, mental health, formative, mobile phone, well-being, apps, quantitative evaluation, real-world data, emotional well-being, pre-post, single arm, quantitative data

## Abstract

**Background:**

With growing evidence suggesting that levels of emotional well-being have been decreasing globally over the past few years, demand for easily accessible, convenient, and affordable well-being and mental health support has increased. Although mental health apps designed to tackle this demand by targeting diagnosed conditions have been shown to be beneficial, less research has focused on apps aiming to improve emotional well-being. There is also a dearth of research on well-being apps structured around users’ lived experiences and emotional patterns and a lack of integration of real-world evidence of app usage. Thus, the potential benefits of these apps need to be evaluated using robust real-world data.

**Objective:**

This study aimed to explore usage patterns and preliminary outcomes related to mental health and well-being among users of an app (Paradym; Paradym Ltd) designed to promote emotional well-being and positive mental health.

**Methods:**

This is a pre-post, single-arm evaluation of real-world data provided by users of the Paradym app. Data were provided as part of optional built-in self-assessments that users completed to test their levels of depression (Patient Health Questionnaire-9), anxiety (Generalized Anxiety Disorder Questionnaire-7), life satisfaction (Satisfaction With Life Scale), and overall well-being (World Health Organization-5 Well-Being Index) when they first started using the app and at regular intervals following initial usage. Usage patterns, including the number of assessments completed and the length of time between assessments, were recorded. Data were analyzed using within-subjects *t* tests, and Cohen *d* estimates were used to measure effect sizes.

**Results:**

A total of 3237 app users completed at least 1 self-assessment, and 787 users completed a follow-up assessment. The sample was diverse, with 2000 users (61.8%) being located outside of the United States. At baseline, many users reported experiencing strong feelings of burnout (677/1627, 41.6%), strong insecurities (73/211, 34.6%), and low levels of thriving (140/260, 53.8%). Users also experienced symptoms of depression (mean 9.85, SD 5.55) and anxiety (mean 14.27, SD 6.77) and reported low levels of life satisfaction (mean 12.14, SD 7.42) and general well-being (mean 9.88, SD 5.51). On average, users had been using the app for 74 days when they completed a follow-up assessment. Following app usage, small but significant improvements were reported across all outcomes of interest, with anxiety and depression scores improving by 1.20 and 1.26 points on average, respectively, and life satisfaction and well-being scores improving by 0.71 and 0.97 points, respectively.

**Conclusions:**

This real-world data analysis and evaluation provided positive preliminary evidence for the Paradym app’s effectiveness in improving mental health and well-being, supporting its use as a scalable intervention for emotional well-being, with potential applications across diverse populations and settings, and encourages the use of built-in assessments in mental health app research.

## Introduction

### Background

Emotional well-being has been widely recognized as a crucial factor in predicting overall health and quality of life [1]. Increased emotional well-being has been robustly associated with a lower risk of both physical and mental health problems, according to numerous studies [2,3]. Research findings from different countries indicate that emotional well-being has been declining within the general population over the last few years [4,5], a trend that was first observed following the beginning of the COVID-19 pandemic in 2020. Since then, problems with well-being and general satisfaction with life have intensified globally, and simultaneously, the demand for easily accessible, affordable, and effective support services has increased [4]. While research suggests that one effective way to enhance emotional well-being is by increasing emotional self-awareness, such a goal is not easily obtainable for many people facing mental health and well-being difficulties. Specifically, conventional face-to-face psychotherapeutic treatments are often inaccessible, usually due to high costs, long waiting times, and inflexible treatment schedules that are not accommodating to people’s daily lives and work-related commitments [4,6]. As a result, mental health apps have been highlighted as a promising solution for delivering these services, given their affordability, ease of access, scalability, and ability to offer anonymous support [6].

In addition, mental health and well-being apps have also begun to be integrated into clinical practice; some therapists use such apps to enhance in-person treatment and provide patients with a tool they can use outside of face-to-face sessions to gain psychoeducational insights, monitor their symptoms, and reflect on their progress [7]. Therapists have expressed positive views about the integration of online mental health tools into their practice, as they can provide a streamlined way to organize and conduct therapy-related activities, improve patient’s accessibility to therapy, and reduce the stigma associated with visiting a mental health professional [8]. Specific types of therapy, such as art therapy, can especially benefit from online mental health tools that enable the creation and sharing of visual arts media, which is a key method to fostering self-expression and well-being for patients [9]. However, concerns have been raised focusing on the high implementation costs associated with the integration of digital apps, both for the therapist and the patient, as well as the privacy, safety, and anonymity of app users, as apps collect sensitive personal information related to patients’ mental and physical health and experiences [10]. In addition, clinicians have expressed some concerns about the robustness of the evidence base behind mental health apps, as some apps’ content and structure are not clearly based on validated therapeutic techniques and have not been investigated in rigorous research studies [10,11]. However, emotional well-being apps have been increasingly studied and have mixed results with effectiveness. Of note, some studies show that, overall, such apps are valued by users for the privacy, personalization, and instant support that they can provide [12].

### Emotional Well-Being

Emotional well-being is a key aspect of mental health that encompasses overall happiness and life satisfaction [13]. Emotional self-awareness, which refers to recognizing and expressing one’s emotions as they occur [14], is crucial for achieving emotional well-being, as low levels of emotional awareness have consistently been linked to diminished emotional well-being, reduced quality of life, problems in interpersonal relationships, and a higher risk of mental health disorders like depression and anxiety [15,16]. In contrast, increased emotional awareness has been linked to better emotional regulation, increased positive emotions and quality of life, improvements in social functioning, and reductions in symptoms of depression and anxiety [17]. Based on these findings, both researchers and policymakers have highlighted emotional well-being as a means to improve overall mental and physical health and reduce the costs linked to poor mental health and the treatment of mental health disorders [1,2].

### Emotional Well-Being Apps

In recent years, a growing number of apps have focused on the connection between emotional self-awareness and emotional well-being has led to a range of digital interventions that focus on this approach [18,19]. These apps, which can be easily accessed on smartphones and tablets, allow users to improve their emotional well-being at their convenience, offering privacy and flexibility, while they are usually either free or have a much lower annual cost than traditional face-to-face therapy [18,19].

A comprehensive review of 52 well-being apps revealed that most of these tools focus on helping users improve their emotional well-being and emotional awareness primarily through techniques such as mindfulness, meditation, cognitive behavioral therapy, mood tracking, and journaling [20]. Mindfulness-based apps, such as Headspace and Calm (Calm.com Inc), have gained popularity by encouraging users to incorporate meditation and breathing exercises into their daily routines. These practices are designed to heighten emotional awareness and help users manage their thoughts and feelings more effectively [21-23]. Similarly, other apps have integrated cognitive behavioral therapy strategies, which are aimed at helping users challenge and reframe negative thought patterns and engage in positive reappraisal [24,25]. Moreover, mood-tracking features in some apps enable users to record and monitor their emotions over time, often providing personalized suggestions for activities that can help regulate and improve their moods [26,27]. This combination of approaches highlights the diverse methods through which digital tools are addressing emotional well-being.

Despite the variety of techniques used, many well-being apps have been critiqued for not fully incorporating lived experiences or storytelling elements in their design [20]. When these elements are included, they typically take the form of fictional narratives or real-life testimonials from individuals who have successfully navigated difficult emotional situations or life events [28]. These storytelling techniques can be powerful, offering users relatable examples and motivating them to engage more deeply with the app’s content [29]. Research indicates that the incorporation of storytelling in therapeutic interventions can improve patient engagement and destigmatize mental health treatment [30]. Effective storytelling has also been shown to enable the delivery of novel health care interventions and capture the unmet needs of patients across demographic strata and treatment areas [31]. However, the limited use of these approaches suggests that there is untapped potential in using storytelling and lived experiences more extensively. In addition, very few apps encourage users to explore their emotional patterns over time. Such exploration could provide valuable insights into their personality traits and behavioral tendencies, offering a deeper understanding of their emotional landscape beyond the immediate moment [20].

Given the rapid growth of well-being apps and the limited research on those that use storytelling, lived experiences, or focus on emotional patterns, there is a clear opportunity for further exploration, which could help identify new strategies to increase user engagement and satisfaction with digital well-being tools, leading to better outcomes in emotional health management. As digital interventions continue to evolve, exploring these areas could play a crucial role in developing more holistic and user-centered tools that better meet the diverse needs of those seeking to improve their emotional well-being [20,32].

### The Paradym App

The Paradym app was created in response to the increasing demand for scalable mental health solutions, driven by global challenges such as the COVID-19 pandemic and increased societal awareness of mental well-being. Paradym addresses these challenges by providing an accessible, evidence-based platform to help users increase their emotional awareness by exploring their emotional patterns, ultimately aiming to enhance emotional well-being, boost self-awareness, and improve overall life satisfaction [32]. The Paradym app introduces users to psychoeducational content through storytelling, covering key aspects of life such as love and relationships, body image, work and success, and identity. In addition, it incorporates elements of lived experience, which involves the user’s personal, first-hand experiences and the significance these experiences bring to their current situations [33].

Paradym’s unique use of emotional pattern recognition and storytelling aims to offer a novel approach within the field of digital mental health and use this combination of lived experience and evidence-based interventions to boost user engagement and emotional awareness. Finally, in contrast to many of the widely used mental health and well-being apps available, Paradym incorporates validated mental health questionnaires in its interface, such as the Satisfaction with Life Scale (SWLS), which allows users to monitor their mood and well-being as they go through the app’s contents. To ensure effective implementation and guide further development, the app’s design considered the principles of the Technology Acceptance Model [34]. This model suggests that users’ engagement with a new digital tool is influenced by their perceptions of its usefulness and ease of use. In addition, the model recommends monitoring user anxiety, as previous research has identified anxiety as an external factor that can impact technology acceptance [35]. A 2021 acceptability and engagement study of the Paradym app [32] revealed that the majority of users found the app easy to use (64%) and used it daily (79%), and post hoc statistical analyses showed a significant increase in the well-being measures (*d*=0.73 for SWLS scores and *d*=0.51 for World Health Organization-5 Well-Being Index [WHO-5] scores) and reduction in symptoms of depression (*d*=–0.38 for Patient Health Questionnaire-9 [PHQ-9] scores) following app use. Importantly, no adverse effects were reported, providing encouraging preliminary evidence for the Paradym app’s effectiveness and usability.

### Aim of This Study

The aim of this study was to expand Paradym’s evidence base by evaluating the app’s effectiveness in addressing symptoms of anxiety and depression and promoting overall well-being and life satisfaction in a real-world context.

## Methods

### Participants and Procedure

Real-world data were collected from an international pool of Paradym app users, who had downloaded and were using the mobile app from the App Store or Google Play. The participant pool for this study consisted of the total of Paradym users who had completed the app’s built-in assessments and questionnaires (Generalized Anxiety Disorder-7 [GAD-7], PHQ-9, WHO-5, and SWLS) at least once. These questionnaires are an optional part of the Paradym app experience, and users can complete them as many times as they wish while using the app to track their progress in their own well-being and life satisfaction. Owing to the fact that assessments are part of the app intervention a specific recruitment procedure was not required to obtain participants’ data for this study. Instead, before completing the questionnaires, all Paradym app users were informed by a written statement that their data would be used for research purposes related to the effects of Paradym on anxiety, depression, general well-being, and satisfaction with life. Users provided consent when signing up to the app and informed that surveys were part of the app user experience. Following the principle of data minimization, users did not provide detailed demographic data beyond the country of residence. The data that informed this study from the in-app assessments was collected between March 2023 and June 2024. Participants did not receive any compensation for filling out the questionnaires as the measures were part of the app intervention.

The Checklist for Reporting Results of Internet E-Surveys (CHERRIES) [36] was used to track the details of this study’s methodology, this provides further detail on recruitment and administration and is provided in in Multimedia Appendix 1.

### Intervention Overview

Paradym is an app designed to promote emotional well-being among the general population, specifically targeting adults aged 18 years and older. Its primary goal is to help users cultivate greater emotional awareness and achieve higher levels of life satisfaction. In its early development, Paradym was funded through crowdfunding, a choice made to avoid the influence of commercial interests during the initial stages of creating the intervention.

Paradym was developed based on evidence-based techniques and was tailored to address themes like success, body, identity, love, and relationships, which were identified by users as important. The platform’s development involved clinical psychologists, coaches, and researchers who collaborated on crafting the content. This included selecting and reviewing psychoeducational materials, appropriate evidence-based strategies, and various activities like journaling and note-taking [32].

The foundation of Paradym is an integrative theoretical approach, enriched by therapeutic strategies like acceptance and commitment therapy and schema therapy, which shape the lived experience and storytelling aspects of the platform. User feedback further refined these approaches, leading to the integration of additional theory-driven strategies to generate content that resonated with the needs expressed by users. The core strategies of Paradym encompass these elements, providing a comprehensive toolkit for personal development.

### Digital Lessons

Paradym’s psychoeducational digital lessons are organized around 5 central themes: awareness, success, love, identity, and body. These areas were selected based on feedback gathered from users during the initial phase of development [32]. The educational content covers several topics, including building emotional awareness, aiding users in recognizing and understanding their emotions, and helping them identify and reinforce their personal values.

Each lesson starts by presenting the educational concept through narratives based on real-life experiences. The use of personal stories is intended to make the material more accessible and relatable, enhance empathy, and encourage users to see themselves in the stories being told. This storytelling approach is known to increase user engagement with both psychological interventions and digital applications. The lessons are available in various formats, including text chapters, audio recordings, and videos, catering to different learning preferences.

### Emotional Patterns

At the conclusion of each digital lesson, users are prompted to examine their own emotional patterns in context with the app’s material, incorporating elements from schema therapy, in order to enhance emotional insight. To facilitate this, the app presents various “emotional patterns” and encourages users to reflect on which pattern they resonate with most at the time of finishing the lesson. This allows users to track and potentially modify their emotional patterns as they progress through the lessons.

### Reflections

Users receive reflections through push notifications, designed to enhance their daily interaction with the app by introducing a new reflection each day. Research has highlighted numerous advantages to such reflection exercises, including aiding individuals in maintaining focus on tasks amidst stressful situations, and reducing tendencies to ruminate, which is often associated with internal struggles and social conflicts. In addition, the use of notifications has been proven to boost user interaction with apps [32], and studies suggest that mobile apps delivering psychological insights are most effective when coupled with proactive user involvement.

### Data Collection Tools

#### Demographics

In order to maintain users’ anonymity and confidentiality, only information regarding their geographic location was collected.

#### Well-Being Measure

The WHO-5 was used to assess well-being in this study. The WHO-5 consists of a brief assessment (under 1 minute) of well-being over a 2-week period [37], in which individuals are asked to indicate how they felt over the past 2 weeks for each of 5 statements using a 6-point Likert scale, ranging from 0=“at no time” to 5=“all of the time.” A higher total score on the WHO-5 indicates a higher level of well-being. The WHO-5 is derived from a 28-item questionnaire based on the Zung scales for depression, distress, and anxiety, as well as from the General Health Questionnaire and the Psychological General Well-Being Scale [38,39]. The WHO-5 has been validated as a measure of depression in both adolescents and older adults, with high measurement invariance [38,39].

#### Satisfaction With Life Measure

The SWLS [40] is a brief (approximately 1 minute) assessment consisting of 5 items designed to measure the global cognitive assessment of satisfaction with life. The SWLS has been shown to have very high construct validity, with Cronbach α=0.85-0.87 [41] and moderately high reliability (Cronbach α=0.78) [42]. A higher score on the SWLS indicates a higher level of life satisfaction.

#### Anxiety Measure

The GAD-7 is a 7-item, brief, widely-used clinical measure that assesses the presence and severity of generalized anxiety disorder. The self-report scale asks how often during the last 2 weeks individuals experienced symptoms of generalized anxiety disorder. Total scores range from 0-21, with cutoff scores of 5, 10, and 15 indicating mild, moderate, and severe anxiety, respectively. Increasing scores on the GAD-7 are strongly associated with greater functional impairment in real-world settings. Sample items are rated from 0 (not at all) to 3 (nearly every day). Scores over 10 have good sensitivity (89%) and specificity (82%) for diagnosis of generalized anxiety disorder by interview, and the scale has high internal reliability, as suggested by Cronbach α=0.92 [43]. The scale has been widely used and considered a valid and reliable screening tool in previous research, presenting good reliability, factorial, and concurrent validity [44]. A higher GAD-7 score indicates more severe anxiety.

#### Depression Measure

The PHQ-9 is a widely used depression scale that scores each of the 9 Diagnostic and Statistical Manual of Mental Disorders, Fourth Edition criteria as 0 (not at all) to 3 (nearly every day). The PHQ-9 has been validated for use in primary care [45]. It is not a screening tool for depression but can monitor the severity of symptoms and response to treatment. Scores over 10 have good sensitivity (88%) and specificity (88%) for the diagnosis of major depression by interview. It has high internal reliability, and Cronbach α=0.89 [46]. The construct validity of the PHQ-9 is also high for community and clinical samples [47]. A high score obtained from the PHQ-9 is indicative of a severe level of depression.

### Data Analysis

Available demographic information (ie, geographic location) and emotional patterns (ie, burnout, sense of thriving, and insecurity) for app users were descriptively analyzed using count and percentages (n, %). Engagement was calculated based on the number of times users visited the app and the length of time the user had been registered on the Paradym app. The results for primary outcomes were largely descriptive in nature, and we evaluated success based on improvements in scores over time. Within-subjects *t* tests were conducted to estimate differences between time 1 (ie, the start of app use) and follow-up. A *P* value of <.001 was considered significant and Cohen *d* estimates were used to measure effect sizes. All statistical analyses were conducted using the SPSS (IBM Corp) software [48].

### Ethical Considerations

This study was conducted with a strong commitment to ethical principles, ensuring the protection of participants’ rights, safety, dignity, and well-being [49]. Data were gathered remotely from individuals who were not part of a clinical setting, and who freely gave their informed consent, allowing their anonymized and nonidentifiable in-app data to be used for research purposes. The research team made their contact information available, offering support and directing participants to additional well-being resources as needed. Participants did not receive any compensation for filling out the questionnaires as the measures were part of the app intervention.

The study was considered to be user testing or service evaluation, and as such, it was not subject to the usual requirements for formal ethical approval by the National Health Service Health Research Authority, aligning with practices in similar studies [50,51]. The certificate of exemption is provided in Multimedia Appendix 2. Since the evaluation included a nonclinical group, it was exempt from registration in a public trials registry, similar to other studies where formal ethical oversight was deemed unnecessary due to the low-risk nature of the research and its nonclinical focus [52,53]. The detailed Terms and Conditions regulations are provided in Multimedia Appendix 3; through these, users of the app agree to their data being used in research.

## Results

### Descriptive Data

A total of 3237 unique users of the Paradym app provided data included in this analysis, between March of 2023 and June of 2024. User uniqueness was determined based on the principal investigator and cookie data. The data were anonymized to protect participants’ identities, so details on their age, gender, and race were not recorded. However, the users had the option to provide information on their geographic location (Table 1).

**Table 1:** Baseline demographic information of Paradym app users whose data were used in this real-world evaluation (N=3237).

Country	Participants, n (%)
United States	1237 (38.2)
Philippines	547 (16.9)
Great Britain	210 (6.5)
Canada	204 (6.3)
Australia	139 (4.3)
Other	900 (27.8)

Users had the option to self-report their perceived levels of burnout, sense of thriving, and level of insecurity before starting to use the app. A total of 1627 users provided information on their level of burnout, and almost half (n=677, 41.6%) reported experiencing strong burnout, while 641 (39.4%) experienced moderate levels of burnout and 309 (19%) experienced low levels of burnout. A total of 260 users rated their sense of thriving, and the majority reported either slight (n=140, 53.8%) or moderate (n=104, 40%) thriving, with only 16 (6.2%) reporting a strong sense of thriving. Among the 211 users who rated their level of insecurity, 34.6% (n=73) and 29.4% (n=62) reported strong and moderate insecurity, respectively, with 36.0% reporting slight insecurity (n=76; Table 2).

**Table 2:** Baseline data on Paradym app users’ levels of burnout (n=1627), thriving (n=260), and insecurity (n=211).

Domain		Participants, n (%)
**Burnout**		
	Slight	309 (19)
	Moderate	641 (39.4)
	Strong	677 (41.6)
**Thriving**		
	Slight	140 (53.8)
	Moderate	104 (40)
	Strong	16 (6.2)
**Insecurity**		
	Slight	76 (36)
	Moderate	62 (29.4)
	Strong	73 (34.6)

### Efficacy Data

**Figure 1:**
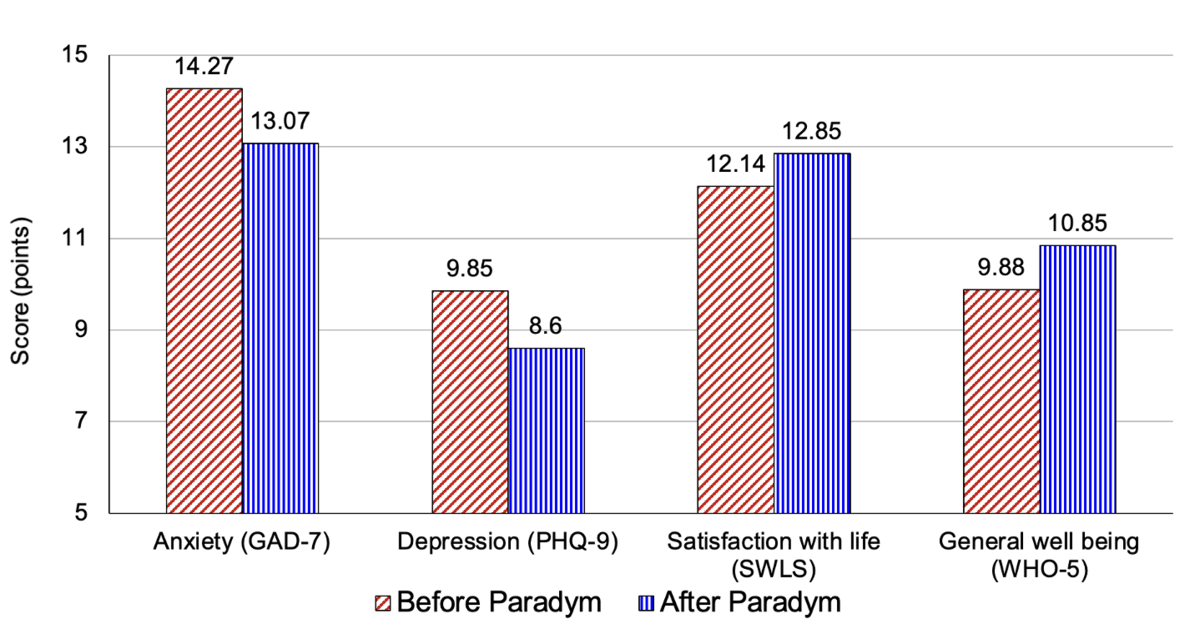
Baseline and follow-up data on participants’ levels of anxiety, depression, satisfaction with life, and general well-being (n=787). GAD-7: Generalized Anxiety Disorder-7; PHQ-9: Patient Health Questionnaire-9; SWLS: Satisfaction with Life Scale; WHO-5: World Health Organization-5 Well-Being Index.

After downloading the app, the first-time users completed questionnaires assessing their levels of anxiety (GAD-7), depression (PHQ-9), life satisfaction (SWLS), and well-being (WHO-5). App usage among the participants in this study varied, but on average, users had been registered for 74 days at the time the data were collected and had visited the Paradym app 10 times, with over 30% of users reporting that they had been using the app somewhat frequently or very often. Of the 3237 app users, just over 20% (n=787) of app users returned to the app more than once to complete mental health and well-being assessments. This was also a significant indicator of those who used the app more frequently (119 times vs 379 times) and had been using the app over a longer period of time (mean 215 days vs mean 36 days). Notably, those who returned to monitor their mental health and well-being indicators at least once showed significant improvements in all 4 mental health and well-being measures. Using available data, within-subjects *t* tests were applied, and the improvement in anxiety (t_521_=5.288, *P*<.001), depression (t_521_=6.667, *P*<.001), satisfaction with life (t_521_=3.191, *P*<.001), and general well-being (t_521_=4.947, *P*<.001) scores were all significant at follow-up (Table 3; Figure 1).

**Table 3:** Baseline (first questionnaire completion) and follow-up (subsequent questionnaire completion) data on Paradym app users’ levels of anxiety, depression, satisfaction with life, and general well-being (n=787).

Outcome measure	Baseline, mean (SD)	Follow-up, mean (SD)	Average baseline to follow-up score change	Effect size (Cohen *d*)
GAD-7^a^	14.27 (6.77)	13.07 (6.63)	1.20	0.231
PHQ-9^b^	9.85 (5.55)	8.6 (5.55)	1.26	0.292
SWLS^c^	12.14 (7.42)	12.85 (7.46)	0.71	0.140
WHO-5^d^	9.88 (5.51)	10.85 (5.5)	0.97	0.217

^a^GAD-7: Generalized Anxiety Disorder-7.

^b^PHQ-9: Patient Health Questionnaire-9.

^c^SWLS: Satisfaction With Life Scale.

^d^WHO-5: World Health Organization-5 Well-Being Index.

## Discussion

### Principal Findings

The aim of this study was to investigate the effects of the well-being app Paradym on the users’ levels of anxiety, depression, satisfaction with life, and general well-being. The findings indicated that users who accessed the app’s contents and completed the built-in mental health and well-being assessments (N=3237) generally reported problems with burnout, insecurity, and a lack of thriving in their lives. In addition, the app’s user base scored highly on scales of depression (PHQ-9) and anxiety (GAD-7) but reported low levels of life satisfaction (SWLS) and well-being (WHO-5). A total of the 787 users chose to complete follow-up assessments of these measures, in which significant decreases in average scores of depression and anxiety were observed. In addition, both life satisfaction and well-being scores increased significantly, providing real-world data on Paradym’s benefits for users with a relatively large sample. However, as this was not a controlled study, it is not possible to make any causal claims or explicitly attribute the findings to the use of the app. Nonetheless, these preliminary findings are overall positive and indicate that Paradym’s digital format allows for wide accessibility and scalability, positioning it as an effective tool for both individual and public mental health strategies.

Despite these positive findings, the number of users who opted to not complete the follow-up mental health and well-being assessments was high (2450/3237), which is commonly observed in studies of mental health and well-being apps [54]. However, this dropout rate only refers to users who did not use the app’s built-in questionnaires. Thus, it is possible that many of these users continued using the app and engaging with its contents, but did not feel the need to assess their progress with the app’s built-in questionnaires. Alternatively, users who experienced higher levels of distress or more severe depression and anxiety symptoms found it more difficult to engage with the app or respond to the outcome measures, as has been observed in other similar studies [55]. In addition, mental health apps generally have high dropout rates, and some studies have found that once users learn a skill or knowledge from a particular app, they stop using it [56]. Further research would be required to understand the factors that influence users to complete the outcome measures beyond the first measure.

### Comparison With Previous Work

This study’s findings on Paradym’s effectiveness in improving mental health and well-being are in line with those observed in studies of other similar apps and add to the growing literature supporting apps’ benefits as mental health tools [57,58].

A significant decrease in symptoms of anxiety and depression has been observed in multiple other studies of popular apps, such as Headspace and Calm, using the same questionnaires as this study [22,59-61]. The same is true for studies examining well-being and life satisfaction [15,62]. In addition, the effect sizes observed for depression and anxiety are consistent with those reported in other studies and meta-analyses of mental health apps [21-23,58,60-63].

However, very few studies have examined the effects that multiple compared to stand-alone assessments or the timing of those assessments could have on the participants, and we did not identify any studies that studied this association using naturalistic data through assessments that were built into the apps’ experience.

### Strengths and Limitations

A key strength of the present analysis rests on its use of a large sample of users under real-world conditions from a diverse user base over a long period of time. Naturalistic data were collected, as users were completing assessments as part of the app experience, and not as part of a controlled study environment. Such analyses could prompt app developers to integrate rigorous data collection processes within their apps’ interface and core features. In addition, the high levels of burnout, insecurity, anxiety, and depression, and low levels of life satisfaction, thriving, and general well-being reported by users at baseline act as a strong indicator that Paradym reached users who were seeking support for their mental health and well-being.

Since no personal data related to the users’ gender, race, or age were collected by the app, in order to ensure users’ anonymity and confidentiality and follow the data minimization principle some limitations in our data analysis are acknowledged. Due to the absence of such detailed demographic data***,*** subgroup analyses based on such characteristics were not conducted as part of this study. Subgroup analyses of effectiveness could provide important insights into how demographic factors affect the outcomes of interest, as has been observed in other studies [64,65].

Further, this study’s single-arm design did not allow for the inclusion of comparators, which could limit the findings’ robustness. Change scores were small but significant so this should be interpreted with caution. The change scores and resulting effect sizes were lower than those commonly accepted to indicate a clinically meaningful difference (approximately 4 points on the GAD-7 [66] and 3 points on the PHQ-9 [67]). However, it should be noted that these thresholds for clinically meaningful change are based on studies of clinical populations, including patients diagnosed with anxiety or depression who had mild to severe symptoms [68]; Paradym is not exclusively aimed at diagnosed patients, and thus, it is expected that the aforementioned clinically meaningful thresholds are not directly applicable to the app’s users, who may have benefited substantially from smaller improvements in questionnaire scores [69].

Finally, while the number of users who dropped out in regards to the questionnaire filling was captured in the data, the exact patterns of app usage were not measured. For instance, some users may have stopped completing the questionnaires, but they maintained a subscription and continued using the app and engaging with its content daily. Collecting more specific data on user engagement patterns would allow for a clearer view of which features of the Paradym app users found more useful, beyond the questionnaires.

### Future Directions

Future studies ought to gain more understanding of the mechanisms underpinning the observed reductions in anxiety and depression symptoms and the increase in life satisfaction and well-being that Paradym users experience. Assessments to capture the specific components of the app’s content and structure that have a greater impact on users could be incorporated into future analyses of user data.

In addition, more detailed information on users’ experiences with burnout, low levels of thriving, anxiety, and depression would help us better understand the needs of the app’s user base, and tailor the app accordingly. For example, questions exploring the specific domains users struggle with (eg, work and personal relationships) could shed light on what kind of content they would benefit the most from (eg, self-assessments, reflection exercises, and guided lessons).

Paradym’s benefits for mental health and well-being could be investigated through randomized controlled trials in the future, in order to understand whether improvements in the users’ mental health and well-being are attributed to ongoing app usage. Comparison groups using similar apps or face-to-face therapy could also be introduced to enhance the results’ validity, robustness, and generalizability.

While not collecting demographic data for this study was a deliberate choice made to preserve the users’ anonymity, future studies could consider collecting data related to age, gender, and race, in order to allow for subgroup analyses of Paradym’s efficacy based on these characteristics. This will be a significant next step in order to answer questions around “what worked, for whom, and under which circumstances.”

Further studies could also explore Paradym’s integration within broader systems, such as health care or educational settings, to evaluate the app’s effectiveness at scale. For instance, patients could be offered access to Paradym if they are placed on a waiting list for in-person therapy, or if they do not feel comfortable initiating face-to-face therapy. In addition, future research could explore the app’s impact across different populations and contexts, including workplace wellness programs or health care partnerships.

Finally, this study’s findings indicate that the anxiety, depression, well-being, and life satisfaction data collection tools incorporated within the app are beneficial to the users. As a result, additional tools, including qualitative data collection measures, could be introduced to the app’s interface, allowing participants to provide valuable and detailed information that could provide insight into their emotional and mental health journey while using the app, as well as a way for them to track their own progress. Feedback measures could also be added to capture the reasons why some participants decide to complete the existing assessments more or less frequently, and why some opt to not complete them at all. This would help to elucidate potential barriers to user engagement with these data collection tools, and whether these are related to the app’s content and structure or to external factors that prevent users from fully engaging with Paradym’s user journey.

### Conclusions

Overall, this analysis highlighted the value of conducting formative research on mental health and well-being apps. The findings suggest that built-in assessments in a mental health app can lead to the collection of valid real-world data from users and promote organic engagement with the app’s features. Among users who completed follow-up assessments in the Paradym app, levels of anxiety and depression decreased significantly, while life satisfaction and general well-being improved significantly. This study provided positive evidence for Paradym’s effectiveness in improving mood and well-being and highlighted the value of the app’s content, functionality, and underlying principles. These encouraging results suggest the scope for further research on Paradym and the integration of built-in assessments in mental health apps.

## Data Availability

The datasets generated and analyzed during this study are available from the corresponding author on reasonable request.

## Appendix

Multimedia Appendix 1 Checklist for Reporting Results of Internet E-Surveys (CHERRIES).

Multimedia Appendix 2 NHS Health Research Authority exemption certificate.

Multimedia Appendix 3 Captures of the Paradym app’s interface, including the onboarding screen, the privacy policy, and the Terms and Conditions.

## Abbreviations

GAD-7: Generalized Anxiety Disorder-7
PHQ-9: Patient Health Questionnaire-9
SWLS: Satisfaction With Life Scale
WHO-5: World Health Organization-5 Well-Being Index
