# Supplementary material for: Improving Mental Health and Well-Being Through the Paradym App: Quantitative Study of Real-World Data
Source: JMIR Form Res. 2025 Jan 23;9:e68031. doi: 10.2196/68031 (PMC11803330; doi:10.2196/68031)
Supplement: Multimedia Appendix 3 [file formative_v9i1e68031_app3.pdf]

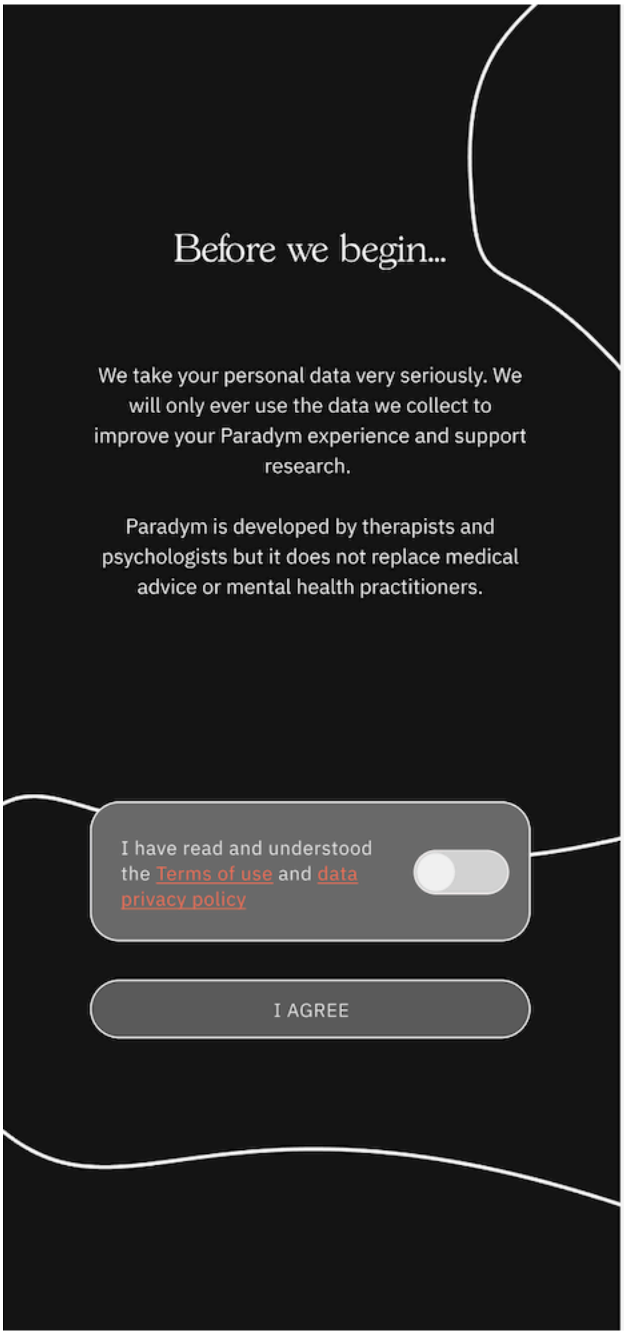
Before we begin...
We take your personal data very seriously. We will only ever use the data we collect to improve your Paradym experience and support research.
Paradym is developed by therapists and psychologists but it does not replace medical advice or mental health practitioners.
I have read and understood the Terms of use and data privacy policy
I AGREE

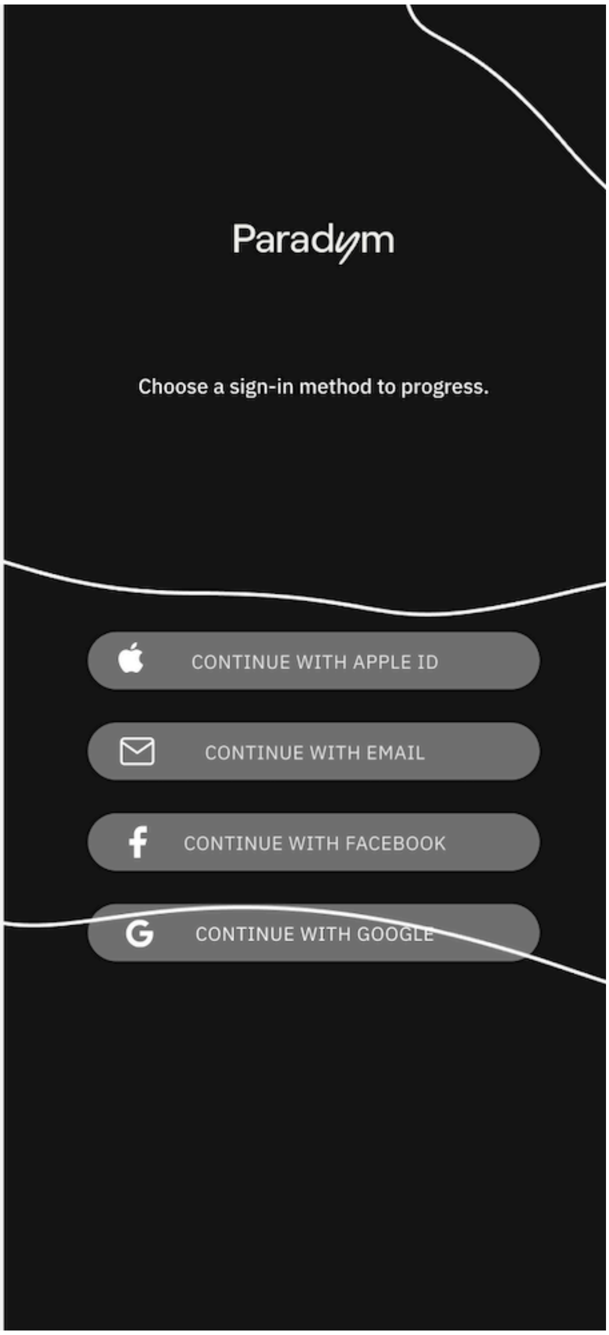
Paradym
Choose a sign-in method to progress.
CONTINUE WITH APPLE ID
CONTINUE WITH EMAIL
CONTINUE WITH FACEBOOK
CONTINUE WITH GOOGLE

4. How Do We Deal With Your Personal Information?

4.1 Your submission of information, including personal data, through or in connection with the Services and our use of cookies is governed by the terms of our privacy policy as updated from time to time, available at https://theparadym.com/privacy-policy/ ("Privacy Policy").

4.2 By using Paradym, you agree that the data collected may be used for research purposes, including academic studies related to mental health and behavioral science. This research is conducted in accordance with our Privacy Policy, and all personal data is anonymized and aggregated to maintain user confidentiality.

What is our “legal basis” for processing your Personal Data?

We want to provide you with the most helpful service we can. In respect of each of the purposes for which we use your Personal Data, the UK GDPR requires us to ensure that we have a “legal basis” for that use. Most commonly, we will rely on one of the following legal bases:

- Where we need to perform a contract (i.e., a subscription) we are about to enter into or have entered into with you (“Contractual Necessity”).
- Where it is necessary for our legitimate interests and your interests and fundamental rights do not

override those interests (“Legitimate Interests“). More detail about the specific legitimate interests pursued in respect of each Purpose we use your Personal Data for is set out in the table below.

We have set out below, in a table format, the legal bases we rely on in respect of the relevant purposes for which we use your Personal Data.

Purpose	Category(ies) of Personal Data involved	Why do we do this	Our legal basis for this use of data
Account Creation	• Identity Data<br>• Contact Data	To register you as a new customer.	Contractual Necessity
To process payments for subscriptions to the Site and/or Application (and their associated services and functionalities, including the Guided Process Programme)	• Identity Data<br>• Contact Data<br>• Finance Data<br>• Transaction Data	To process and deliver your order including manage payments, fees and charges.	Contractual Necessity
Fraud Prevention	• Identity Data<br>• Contact Data	To keep the Site and/or Application, our services and associated systems operational and secure.	Legitimate Interests. We have a legitimate interest in ensuring the ongoing security and proper operation of our services, Site and associated IT services and networks.
Troubleshooting	Technical Data	To track issues that might be occurring on our systems.	Legitimate Interests. It is in our legitimate interests that we are able to monitor and ensure the proper operation of our Site and/or Application and associated systems and services.
Marketing	• Identity Data<br>• Contact Data<br>• Marketing Data	To form a view on what we think you may want or need, or what may be of interest to you. This is how we decide which services and offers may be relevant for you.	Legitimate Interests. We have a legitimate interest in providing you with updates on our Site and/or Application and related offers where you have purchased or shown interest in similar services from us.
Research	• Mental Health Data	• To provide you with a 'Wellbeing Score' from time to time to enable you to track your progress.<br>• To contribute to the advancement of mental health studies and better understand user behaviour for the improvement of your app experience. All data is aggregated and anonymised to protect your privacy.	Consent & Legitimate Interests. We have a legitimate interest in using the data we collect to improve the Paradym experience, conduct mental health research, and advance scientific understanding in the field. This research is essential to enhancing the effectiveness of our services, understanding user behaviour, and contributing to academic studies on mental health.
